# Plasmonic detection and visualization of directed adsorption of charged single nanoparticles to patterned surfaces

**DOI:** 10.1007/s00604-016-1956-7

**Published:** 2016-10-04

**Authors:** Vitali Scherbahn, Shavkat Nizamov, Vladimir M. Mirsky

**Affiliations:** Nanobiotechnology – Institute of Biotechnology, Brandenburgische Technische Universität Cottbus – Senftenberg, 01968 Senftenberg, Germany

**Keywords:** Surface plasmon microscopy, Self-assembled monolayers, ω-functionalized alkyl thiols, Selective adsorption, Sensor array, Surface charge, Nanoparticle imaging

## Abstract

It has recently been shown that surface plasmon microscopy (SPM) allows single nanoparticles (NPs) on sensor surfaces to be detected and analyzed. The authors have applied this technique to study the adsorption of single metallic and plastic NPs. Binding of gold NPs (40, 60 and 100 nm in size) and of 100 nm polystyrene NPs to gold surfaces modified by differently ω-functionalized alkyl thiols was studied first. Self-assembled monolayers (SAM) with varying terminal functions including amino, carboxy, oligo(ethylene glycol), methyl, or trimethylammonium groups were deposited on gold films to form surfaces possessing different charge and hydrophobicity. The affinity of NPs to these surfaces depends strongly on the type of coating. SAMs terminated with trimethylammonium groups and carboxy group display highly different affinity and therefore were preferred when creating patterned charged surfaces. Citrate-stabilized gold NPs and sulfate-terminated polystyrene NPs were used as negatively charged NPs, while branched polyethylenimine-coated silver NPs were used as positively charged NPs. It is shown that the charged patterned areas on the gold films are capable of selectively adsorbing oppositely charged NPs that can be detected and analyzed with an ~1 ng⋅mL^−1^ detection limit.

**Electronic supplementary material:**

The online version of this article (doi:10.1007/s00604-016-1956-7) contains supplementary material, which is available to authorized users.

**Graphical abstract:**
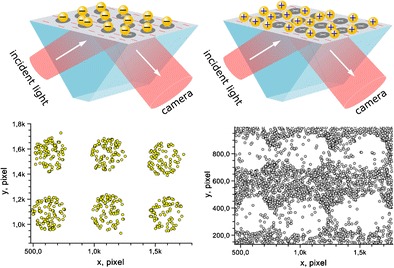
Self-assembled monolayers of ω-functionalized alkyl thiols were deposited on a gold layer of a patterned sensor array. The charge-selective binding of single nanoparticles to such surfaces was registered by wide-field surface plasmon microscopy.

## Introduction

Detection, quantification, and characterization of nanoparticles of biological origin or engineered nanomaterials is the actual challenge of different fields of analytical science, including food and environmental safety, bioanalytics, and medical diagnostics [1–4]. Several methods to detect single nanoparticles (NPs) were reported [5]. One of such techniques is based on surface plasmon resonance (SPR) which belongs to the highly sensitive refractometric transducers [6] and has become a routine technique for investigation of interactions of biomolecules [7, 8]. This approach has been also realized as an imaging system (SPR imaging or SPM – surface plasmon microscopy) [9].

The sensitivity of SPR devices is limited by fluctuations of refractive index of aqueous environment [10, 11] and can be increased by corresponding referencing. This referencing was performed using the measurements at two different wavelengths [12, 13] or at closely placed sensing and referencing spots [14, 15], while the mathematical procedure was realized using image analysis [14], electrical [12] or optical [15] subtraction. It has been recently demonstrated that adsorption of NPs to the resonant layer of SPR sensors provides well measurable signals. A high sensitivity of this approach is also based on the referencing: a few μm vicinity around these NPs in course of their adsorption is compared with the rest of the sensor surface.

Two SPM approaches for detection of single nanoparticles have been developed [5, 16–20]. Both are based on the excitation of surface plasmons in Kretschmann configuration. But the optical system used for coupling and imaging of the sensor surface under conditions of plasmon resonance can be implemented either by using of a high-numerical aperture (high-NA) microscope objective [5, 16, 17] or by using separate objectives and camera tilted according to Scheimpflug principle [18–20]. The high-NA approach allows one to acquire highly resolved low distortion images but leads to the limitation of the field of view, which is in this case typically smaller than 100 μm × 80 μm (< 0.01 mm^2^). Using this approach, metallic and organic nanoparticles have been detected and binding of DNA modified gold nanoparticles to the surface coated by a complementary DNA sequence have been shown [21]. The technique can be used also in air [22, 23] where the difference in the refractive index between analyte and environment is much higher. It was also applied for detection of viruses [24] and for tracking of mitochondria in the living cells [25].

**Fig. 1:**
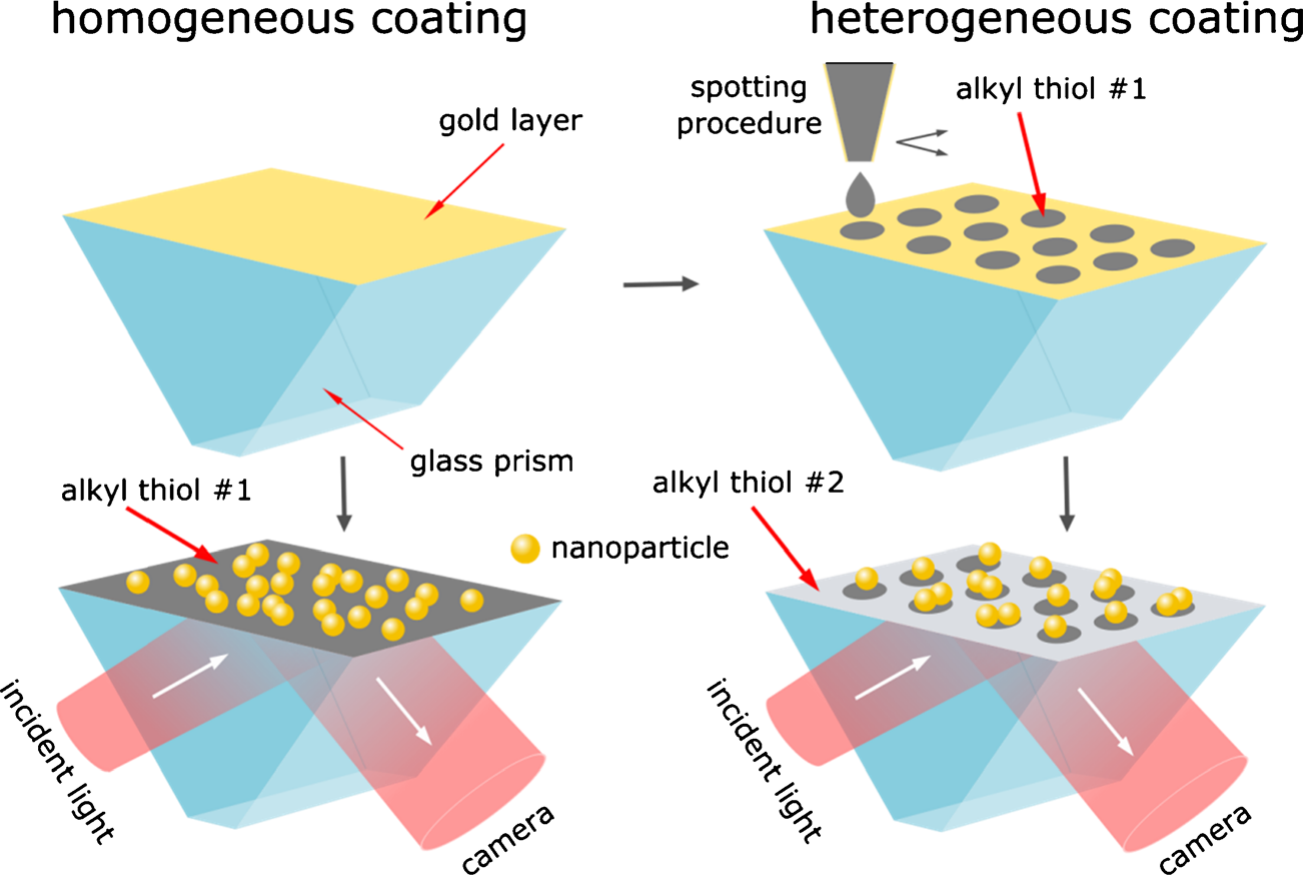
Self-assembled monolayers of ω-functionalized alkyl thiols were deposited on the gold layer as a homogeneous coating (*left panel*) or as patterned layers consisting of two types of ω-functionalized alkyl thiols (*right panel*). The binding of nanoparticles to such surfaces was detected by wide-field surface plasmon microscopy

Another approach, contrarily to the high-NA approach, is based on the conventional SPM [9] where the incident light is coupled to a plasmonic sensor layer by a glass prism (Fig. 1). This approach leads to a lower numerical aperture of this optical system and to a correspondingly lower optical resolution. However, the sensitivity is still sufficient for detection of single nanoparticles. Such a configuration has been used for detection of single polystyrene nanoparticles (PS NPs) and HIV virus-like particles (HIV-VLP) [18, 19]. Despite the lower spatial resolution, this configuration has an important advantage: the monitored (imaged) surface is much larger - typically few mm^2^. Therefore, this approach has a higher dynamic range and lower limit of detection – starting from below 10^6^ NPs⋅mL^−1^ up to 10^10^ NPs⋅mL^−1^ thus covering the whole ppb concentration range [26, 27]. The large sensor surface also provides more possibilities for the investigation of the binding of NPs to different surfaces. Moreover, it can be used for deposition of different receptors and for formation of sensor arrays; this is demonstrated in the present work.

We report here a comparative investigation of interaction of citrate stabilized gold nanoparticles (cit-Au NPs) and sulfate-terminated polystyrene nanoparticles (sPS NPs) with various surfaces formed by self-assembly monolayers (SAM) of ω-functionalized alkyl thiols. First, the influence of ionic strength and surface coatings on the binding of cit-Au NPs and sPS NPs was studied. Then, the binding of nanoparticles to the surface patterned by deposition of spots with different ω-functionalized alkyl thiols was registered. The direct detection and visualization of adsorption of single NPs to the differently functionalized sensor surfaces enables ultrasensitive and unambiguous characterization of NP-surface interaction. A brief schematic illustration of the study is depicted in Fig. 1. Negatively charged cit-Au NPs and sPS NPs, and positively charged branched-polyethylenimine-coated silver nanoparticles (bPEI-Ag NP) were applied. The results indicate that the electrostatic interaction is essential for a selective binding of NPs. Therefore, the surfaces patterned by oppositely charged ω-functionalized alkyl thiols can be used as sensors to determine the sign of the surface charge of nanoparticles.

## Materials and methods

### Materials

11-Mercaptoundecanoic acid (C10-COOH), (11-Mercaptoundecyl)tetra(ethylene glycol) (C11-(EG)_4_-OH), 1-Undecanethiol (C10-CH_3_), 11-Mercaptoundecyl amine hydrochloride (C11-NH_2_), were purchased from Sigma Aldrich. 11-(Mercaptoundecyl)trimethylammo-nium chloride (C11-N^+^(CH_3_)_3_ Cl^−^) was purchased from ProChimia Surfaces (www.prochimia.com), 100 nm latex beads (sulfate-terminated polystyrene nanoparticles, sPS-NPs) and citrate stabilized gold nanoparticles (20 nm, 40 nm, 60 nm and 100 cit-Au NPs) from Sigma Aldrich (www.sigmaaldrich.com), sodium citrate from Sigma-Aldrich, 99.9 % ethanol, boric acid and phosphate salts from Merck (www.merck.de), dimethyl sulfoxide (DMSO) and sodium chloride (NaCl) from Roth (www.carlroth.com). Branched-polyethylenimine-coated 60 nm silver nanoparticles (bPEI-Ag NP) were purchased from nanoComposix (www.nanocomposix.com). All solutions were prepared using deionized water additionally purified by ELGA-Classic system (elgalabwater.com). All suspensions of nanoparticles were diluted to a final concentration of ~10^8^ NP⋅mL^−1^.

The control measurements of hydrodynamic size and ζ-potential of nanoparticles were performed at pH 5 (1 mM citrate), pH 7 (1.2 mM phosphate), and pH 9 (1 mM boric acid) using Malvern Zetasizer Nano ZS (www.malvern.com).

### Functionalization of sensor surface

Prior to functionalization, the gold coated sensor prisms were cleaned for ~20 s by freshly prepared “piranha solution” (1:3 v: v mixture of 32 % H_2_O_2_/H_2_SO_4_), rinsed thoroughly with water and ethanol, and dried at room temperature. *Caution: piranha solution reacts violently with most organic materials and must be handled with extreme care.* In the next step, the cleaned prisms were put in 1 mM ethanolic solution of the corresponding ω-functionalised alkyl thiol, and were incubated overnight at room temperature. Before usage, the prisms were rinsed thoroughly by ethanol, and were dried by air.

The patterned C11-N^+^(CH_3_)_3_ Cl^−^ / C10-COOH surfaces were prepared in two steps. First, 10 mM of C11-N^+^(CH_3_)_3_ Cl^−^ solution in DMSO were deposited by a non-contact dispenser sci-FLEXARRAYER-S3 (www.scienion.com) with a dot pitch of 200–300 μm forming an array of 100–250 μm spots. After 20 min incubation in DMSO atmosphere at room temperature, the spotted gold surface was rinsed thoroughly by DMSO and by ethanol followed by incubation in ethanolic solution of 1 mM C10-COOH for 10 min - at this step, the rest of the gold area is functionalized [15]. Due to very slow exchange kinetics of alkyl thiols [28, 29] we assume that this procedure does not lead to any essential modification of the formerly coated spots by C11-N^+^(CH_3_)_3_ Cl^−^ alkyl thiol. Then, the prism was rinsed by ethanol, dried, and mounted in the SPM device.

### Surface plasmon microscopy setup

The setup for SPM was developed within the EC-FP7 project “Nanodetector” (www.nanodetector.eu). 642 nm SM-fiber coupled laser diode with current and temperature controllers (LP642-SF20, LDC205C and TED200C correspondingly) were from Thorlabs (www.thorlabs.com). Light was collimated by the 16 mm focus length objective (MVL16, Thorlabs) and directed through 14 mm free aperture Glan polarizer (EksmaOptics, www.eksmaoptics.com) set to the *p*-polarisation (with regard to the gold coated prism surface). Gold coated sensors consist of SF - 10 (*n* = 1.72) glass prisms with 43–45 nm gold layer on 3–5 nm titanium adhesive layer.

The slope of the SPR curve and correspondingly the absolute value of the signal of the SPR reflected light shows its maximum at approximately 0.3–0.5 of the SPR reflectivity [30]. However, taking into account the relative changes (the ratio of signal changes to the mean signal value), the highest signal-to-noise ratio is expected at the angle much closer to the SPR minima [31]. Moreover, in close vicinity to SPR minimum the reflected light intensity tends to zero which makes more affordable a registration of small changes caused by adsorption of nanoparticles. Therefore, the measurements were performed at the angle 0.1–0.3 degrees before SPR minimum.

The image was formed on a MT9P031 monochrome CMOS image sensor. The image sensor has a 2592 × 1944 pixels resolution with a pixel size of 2.2 μm. The images with field of view of about 1.3–1.5 mm^2^ were read at ~15 frames per second at full resolution by the Beagleboard-XM single-board computer, averaged over 16 consecutive frames and recorded by PC for further analysis by the homemade software. Initial version of this software was reported in [23] whereas the advanced version based on cluster analysis of images and template matching algorithms was presented in [27].

### Measurement sequence

The sample suspension was pumped by a solenoid-operated micro-pump (Biochem Valve, 130SP1220-1TP, 12 V–DC, www.biochemfluidics.com) at a flow rate of 1 mL⋅min^−1^. The measurement was started by calibration of the system in the units of the refractive index; it was performed by pumping of a 1.5 mL pulse of the background solution containing additionally 20 mM NaCl. After a washing step, 1.5 mL suspensions of each type of nanoparticles (40 nm, 60 nm, 100 nm cit-Au NPs and 100 nm sPS NPs) were pumped through the flow cell with a washing step in between by 1.5–5 mL of the background solution. The investigation of NPs binding on homogeneously modified surface was performed in 1.2 mM phosphate buffer with and without 200 mM NaCl at pH 7. The calibration was performed in the corresponding buffer containing 20 mM NaCl or 180 mM NaCl (in case of using 200 mM NaCl in the background solution). When using patterned surfaces, no calibration step was performed and, additionally, 60 nm bPEI-Ag NPs suspended in pure water were investigated.

## Results and discussion

### Characterization of nanoparticles

As the work was performed with commercial nanoparticles, their characterization was limited to measurements of ζ-potential and hydrodynamic diameter. The measurements were performed using dynamic light scattering. As shown in Table 1, the obtained size values are very close to the data of supplier: the deviation for “100 nm” NPs was below 10 %. No statistically significant changes of their measured size were observed after an increase of the ionic strength by the addition of 200 mM NaCl or after 1-day incubation at room temperature.

**Table 1:** ζ-potential and hydrodynamic size of sulfate-terminated polystyrene nanoparticles (sPS NPs) and citrate stabilized gold nanoparticles (cit-Au NPs) at different conditions

Buffer composition			sPS NPs		cit-Au NPs	
Buffer	[NaCl] *mM*	pH	ζ-potential, mV	Hydrodynamic diameter, nm	ζ potential mV	Hydrodynamic diameter, nm
1 mM citrate	0	5	-23.5 ± 1.9	N/A	-29.5 ± 0.6	N/A
1.2 mM phosphate	0	7	-47.7 ± 5.0	93.9 ± 1.4	-37.2 ± 0.9	109.9 ± 0.6
1 mM boric acid	0	9	-27.4 ± 1.8	N/A	-45.8 ± 2.6	N/A
1 mM citrate	200	5	-7.4 ± 0.3	N/A	-5.7 ± 0.9	N/A
1.2 mM phosphate	200	7	-23 ± 1.7	98.5 ± 4.1	-8.7 ± 0.8	109.3 ± 0.3
1 mM boric acid	200	9	-17.7 ± 1.5	N/A	-11.4 ± 0.6	N/A

An increase of pH from 5 up to 9 leads to a monotonous change in ζ-potential of cit-Au NPs in 1 mM buffer without NaCl with values about −30 mV to −46 mV (Table 1). In the presence of 200 mM NaCl the absolute value of the ζ-potential decreases down to the range between −6 mV and −11 mV. It can be explained by electrostatic screening where in the presence of 200 mM NaCl the Debye length decreases from 3.6 nm down to 0.7 nm. The surface charge of sPS NPs is determined by sulfate groups which are completely deprotonated in the used pH-range. The observed non-monotonous dependence of the ζ-potential on pH can be explained by adsorption of phosphate ions from the phosphate buffer. Notably, the relatively low values of ζ-potentials at 200 mM NaCl did not lead to aggregation of nanoparticles.

### Visualization of nanoparticles on the surface

Once a nanoparticle has bound on the sensor surface, it causes a weak optical signal, which can be visualized only after some preprocessing [23]. The crucial step of this analysis is the formation of the differential record acting as a dynamic correction of the background and providing a possibility to compensate not only the static background signal but also some background drift. The result of this operation is the intensity change (differential frame) between two subsequent frames caused mainly by adsorbed (or desorbed) NPs. Therefore the number of images of NPs in each differential frame characterizes the number of NPs adsorbed to (or desorbed from) the surface during the time between two subsequent frames which is ~1.1 s in full resolution.

The penetration depth of evanescent field into aqueous environment is about 200 nm for 650 nm wavelength [12]. Therefore, we can expect that we detect NPs on the surface or at the distance till ~200 nm from the surface. The traveling speed of NPs in aqueous suspension depends on their size; 1.6 m⋅s^−1^ for 10 nm NPs and 5.2 × 10^−2^ m⋅s^−1^ for 100 nm NPs [32]. Considering the penetration depth of the evanescent field for the 642 nm laser wavelength as ~200 nm, the traveling time of NPs within this region is ~0.1 μs and 4 μs, for 10 nm and 100 nm NPs, respectively, if these NPs are moving perpendicular to the surface. This time is much shorter than the time between subsequent averaged frames, which is ~1.1 s. Therefore, we cannot expect well measurable signals from NPs bouncing from the surface.

**Fig. 2:**
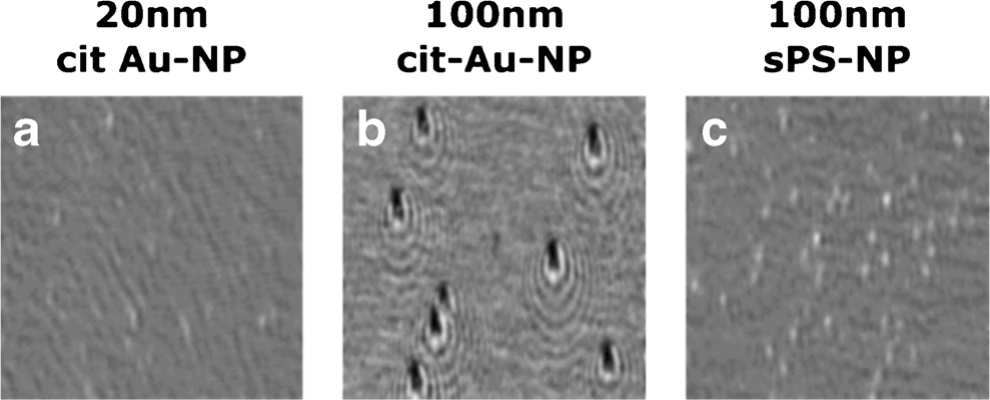
Differential images of nanoparticles on the surface coated by C11-(CH_3_)_3_^+^ Cl^−^ alkyl thiol. 20 nm (**a**), 100 nm citrate stabilized gold nanoparticles (**b**), and 100 nm sulfate-terminated polystyrene nanoparticles (**c**) at concentrations ~5 × 10^8^ nanoparticles⋅mL^−1^, except 10^8^ nanoparticles⋅mL^−1^ for 100 nm citrate stabilized gold nanoparticles. Image size: 300 X 300 pixels (~ 0,02 mm^2^)

The size and shape of the obtained images of adsorbed NPs is the result of complicated interference of the incident, reflected, and diffracted light. These images do not correspond to the real size and shape of NPs and look like a dark or bright oval spot or more complex shapes surrounded by one or more ovals (Fig. 2). Their shape is very close to that obtained by theoretical calculations [33, 34].

It is logical to expect that the differential images of the adsorbed and desorbed NPs look like photographic “positives” and “negatives”. Therefore, the detailed quantitative analysis of the number and time dependent evolution of these images allows one to get quantitative information on adsorption and desorption of NPs to/from the surface [26, 27]. As single NPs are detectable, their minimal adsorption rate corresponds to one NP per reasonable measurement time. The adsorption rate of one NP per second per total sensor area corresponds to ~10^6^ NPs⋅mL^−1^ or ~1 fM. The optimal concentration range for the detection technology was found to cover the whole ppb range (1–1000 ng⋅mL^−1^) [27]. An increase of the measurement time and/or sensor area lead to further improvement of the detection limit.

### Detection of single nanoparticles on the surfaces homogeneously coated by self-assembled monolayers

In order to identify the most suitable SAMs for creating heterogeneously patterned sensor surfaces, we first monitored the adsorption behavior of cit-Au and sPS NPs on homogeneously coated surfaces. Additionally, we investigated the influence of the higher ionic strength (salinity) on the adsorption of these NPs. For coatings, five types of ω-functionalized alkyl thiols with different terminal moieties were selected: C11-NH_2_, C11-N^+^(CH_3_)_3_ Cl^−^, C10-COOH, C11-(EG)_4_-OH and C10-CH_3_. 20, 40, 60, 100 nm cit-Au NPs and 100 nm sPS NPs were used. All types of NPs showed a negative value of ζ-potential (Tab. 1) caused by citrate adsorbed on gold NPs or by sulfate groups of plastic NPs.

**Fig. 3:**
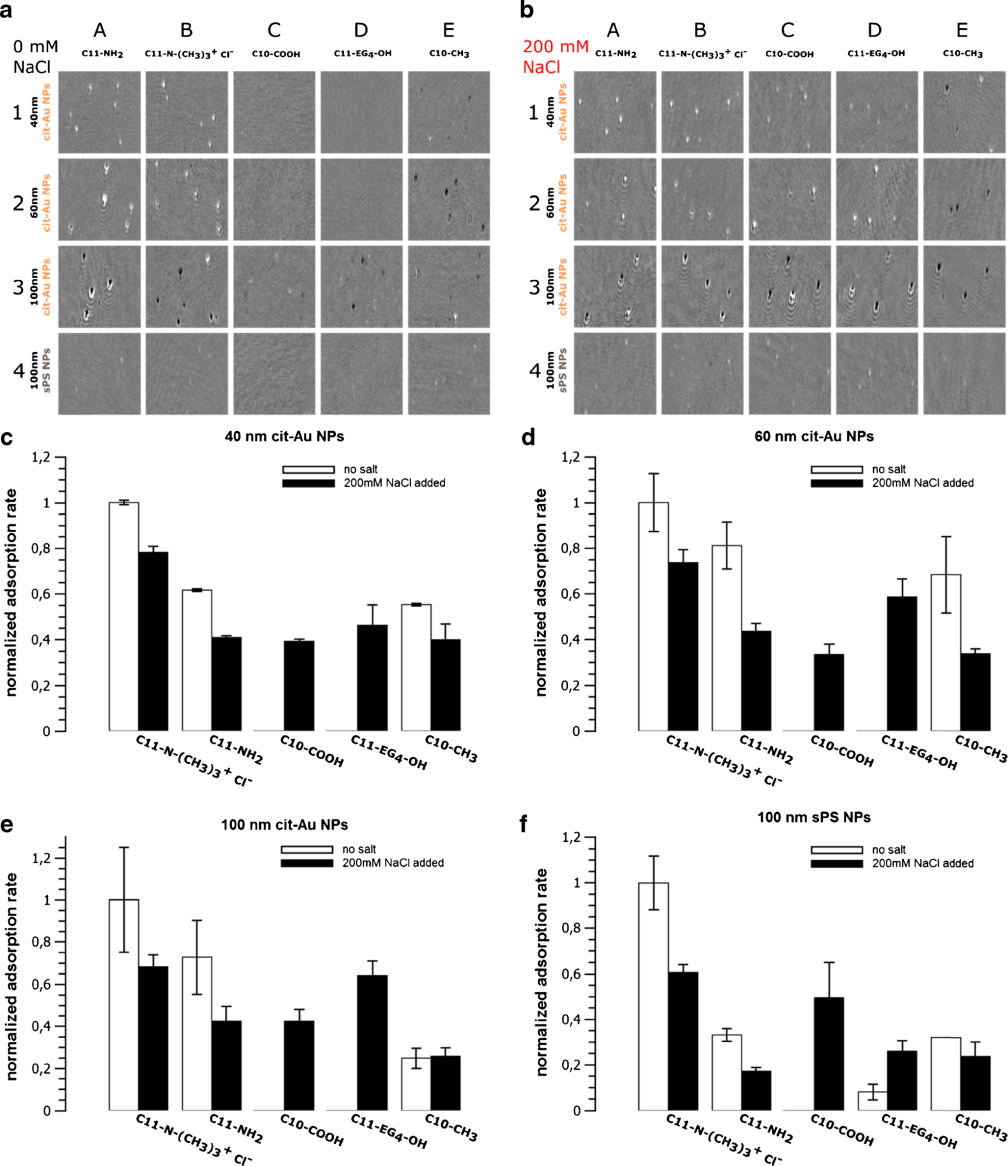
Differential SPM images of nanoparticles adsorbed on the sensor surface coated by C11-NH_2_ (A), C11-N^+^(CH_3_)_3_ Cl^−^ (B), C10-COOH (C), C11-(EG)_4_-OH (D) and C10-CH_3_ (E) - alkyl thiols. Detection of nanoparticles without (**a**) and with (**b**) 200 mM sodium chloride and their corresponding adsorption rates at a concentration of 10^8^ nanoparticles⋅mL^−1^ (**c** – **f**). The SPM images are 300 px X 300 px cutouts (~ 0.02 mm^2^) from the full visible sensor area (2592 px X 1944 px, ~1.3 mm^2^)

Adsorption of these nanoparticles was studied at pH 7 at low ionic strength (Fig. 3a) and in the presence of 200 mM NaCl (Fig. 3b). Quantitative comparison based on normalization to C11-N^+^(CH_3_)_3_ Cl^−^ coated surface possessing the highest adsorption rate, is shown in Fig. 3 c-f. The values of the Debye lengths in the buffer solutions with- and without 200 mM NaCl are 3.6 nm and 0.7 nm, correspondingly. Thus, at low ionic strength a strong adsorption of all studied nanoparticles to the positively charged surface formed by C11-NH_2_ and C11-N^+^(CH_3_)_3_ Cl^−^ was observed (Fig. 3a, A-B). Oppositely, no binding of sPS or 40 and 60 nm cit-Au NPs to the negatively charged surfaces formed by C10-COOH (Fig. 3a, C) occurred. This indicates clearly the role of electrostatic interaction in the adsorption of nanoparticles.

Unlike on C11-NH_2_ and C11-N^+^(CH_3_)_3_ Cl^−^ coatings, the behavior of 100 nm cit-Au NPs on C10-COOH and C11-EG_4_-OH coatings was quite different: it was possible to observe some weak signals corresponding to two types of images, while one of these images looked like a negative of another one. The magnitude of these signals is much lower than in the case of adsorption to the two positively charged surfaces. Probably, in this case we detect NPs which adsorb/desorb from the surface within one frame. The shortness of NPs’ stay on the surface and the exponential decay of their image intensity with the distance to the sensor lead to much weaker signals compared to usual signals from firmly adsorbed NPs. In the case of smaller NPs, such transient signals are not measurable.

In the absence of specific adsorption of sodium and chloride ions, the addition of 200 mM NaCl leads to a decrease of the Debye length down to 0.7 nm and of the absolute values of the surface potentials. Therefore, one can expect a decrease of electrostatic interaction and a subsequent decrease of adsorption of negatively charged NPs to the positively charged surfaces but also a decrease of electrostatic repulsion of these NPs from the negatively charged surface. Such effect was really observed for C11-N^+^(CH_3_)_3_ Cl^−^ and C11-NH_2_ coated surfaces. In this case, a decrease of the number of adsorbed NPs was observed (Fig. 3 c-f). Notably, in the presence of 200 mM NaCl, a strong adsorption of NPs to the C10-COOH coated surfaces was observed. Therefore, a decrease of the electrostatic barrier leads to adsorption caused by other types of interactions (i.e., Van der Waals attraction). The same adsorption behavior of NPs is observed for the C11-EG_4_-OH coated surface, but in this case the physical nature of the energetic barrier is rather unclear.

Direct electrostatic interaction is not expected for C10-CH_3_ coating. According to [35], such surfaces can adsorb OH^−^ ions, but the observed adsorptive properties of these surfaces (Fig. 3 c-f) are more typical for the surfaces which are positively charged.

The observed influence of the surface coating indicates that the process is not completely limited by diffusion. We cannot exclude some contribution of gravity into binding of NPs, but the observed influence of the surface coating indicates that this effect is not very strong. An attempt to separate a contribution of the adsorption and of the diffusion processes into the observed adsorption rate will be presented elsewhere.

The results demonstrate that the surface coated by C11-N^+^(CH_3_)_3_ Cl^−^ terminated alkyl thiol provides the most effective binding of negatively charged nanoparticles. Such coating type can be used as an unselective one for detection of nanoparticles; namely, this coating was applied in our recent work focused on detection and characterization of nanoparticles in complex media [27]. Albeit few times weaker, the adsorption of nanoparticles to the C11-EG_4_-OH coated surface was observed at high ionic strength; thus indicating that such a surface cannot be reliably applied in analytics of NPs as a negative control.

### Directed binding of single nanoparticles to the patterned surfaces

**Fig. 4:**
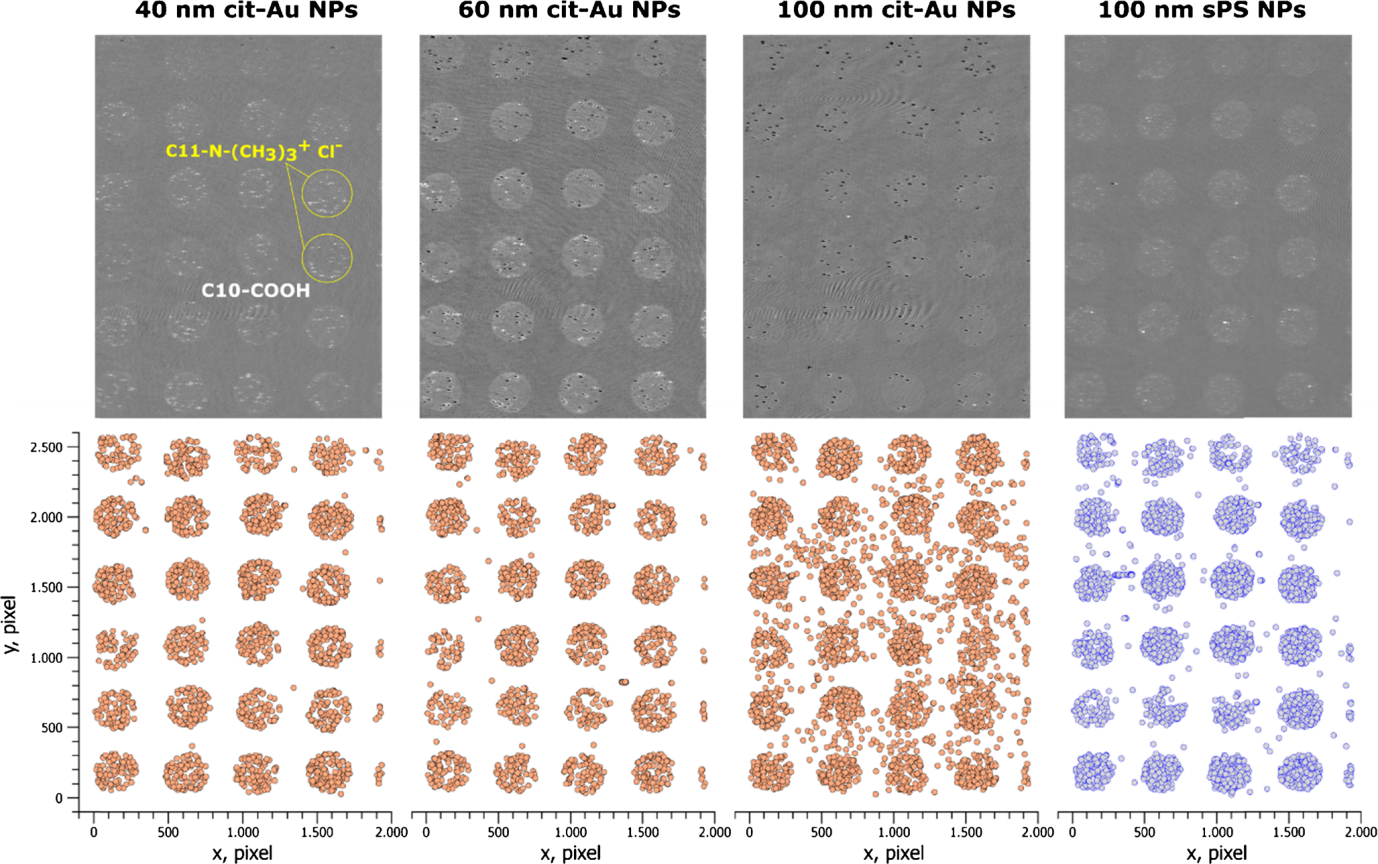
Visualization of selective adsorption of negatively charged nanoparticles without addition of sodium chloride on the surface coated by spots with C11-N^+^(CH_3_)_3_ Cl^−^ (120–130 μm spot diameter) surrounded by a coating with C10-COOH alkyl thiol. Upper panel: Differential SPM images. Bottom panel: Visualization of nanoparticles adsorbed within 90 s. Visible area is ~1.3 mm^2^

**Fig. 5:**
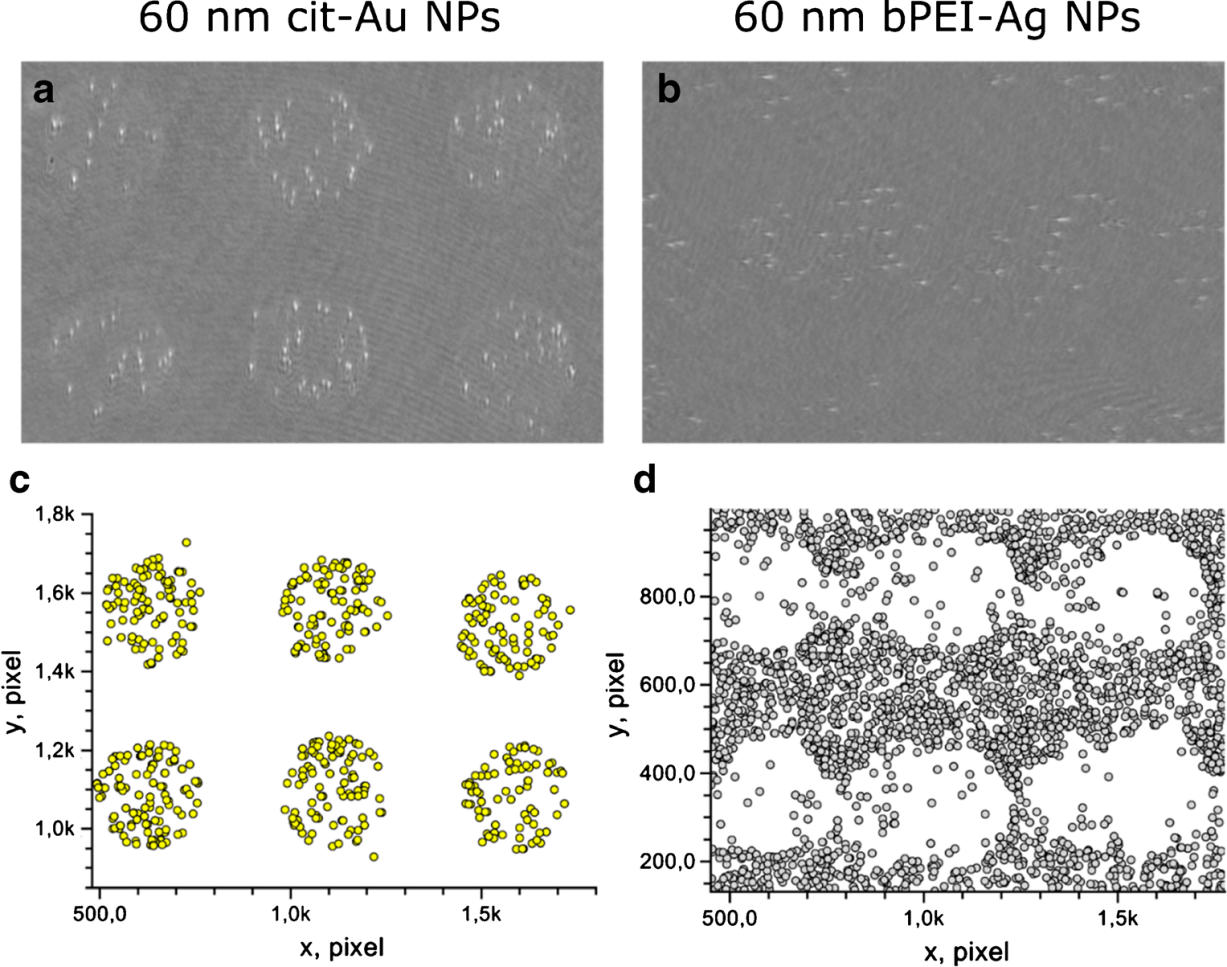
Visualization of selective adsorption of negatively charged citrate stabilized gold nanoparticles (left pannel) and positively charged branched-polyethylenimine coated silver nanoparticles (right pannel) without addition of sodium chloride on the surface coated by spots with C11-N-(CH_3_)_3_^+^ Cl^−^ (120–130 μm spot diameter) and C10-COOH coated area in-between. Differential SPM images (a-b). Visualization of single nanoparticles adsorbed within 90 s (c-d). Visible area is ~0.3 mm^2^

The highest adsorption rates of negatively charged cit-Au NPs and sPS NPs are observed for the C11-N^+^(CH_3_)_3_ Cl^−^ coating whereas the lowest adsorption rates were observed for C10-COOH coated surface at low ionic strength (Fig. 3 c-f). These two coatings, providing maximal contrast for binding of NPs, were selected for surface patterning. Using a non-contact piezoelectric dispenser, droplets of N^+^(CH_3_)_3_ Cl^−^ terminated alkyl thiol were deposited to the gold surface. Then, the space between droplets was coated by C10-COOH alkyl thiol. The results of adsorption of different negatively charged NPs to such patterned surface are shown in Fig. 4 and in Video1 (Supplementary material), while the adsorption of the positively charged bPEI-Ag NPs is presented in Fig. 5 and in Video2 (Supplementary material). The measurements were performed at low ionic strength. A clear selectivity of adsorption of all negatively charged NPs to the C11-N^+^(CH_3_)_3_ Cl^−^ coated spots was observed. Almost no binding of 40- and 60 nm cit-Au NPs to the surface coated by C10-COOH was observed, while this selectivity was some lower for sPS NPs and large cit-Au NPs, probably due to the contribution of the gravity. Similar results were also obtained for patterned surfaces containing spots with C16-NH_2_ and the rest area with C10-COOH.

The behavior of the positively charged bPEI-Ag NPs showed an opposite effect: they adsorbed to the negatively charged C10-COOH coated part of the sensor surface (Fig. 5). The results demonstrate that SPM in combination with the patterned surfaces coated by ω-terminated alkyl thiols with oppositely charged functional groups can be used to distinguish positively and negatively charged NPs.

## Conclusion

Wide-field SPM is the real-time label-free optical method for detection of NPs at ppb concentration range. We have applied it for investigation of binding of single nanoparticles to sensor surfaces coated by differently ω-functionalized alkyl thiols. The observed difference in the adsorption rates to various surfaces indicates the possibility to develop sensor arrays for analysis of nanoparticles. The concept was proved using the simplest array consisting of patterns with positively charged spots whereas the rest area was coated by negatively charged alkyl thiols. A strong difference in binding of charged nanoparticles to the oppositely charged parts of the surface was observed. This effect can be used for separation of NPs based on the difference in their charge. Additional parameters, which can be used to control the binding of nanoparticles to surfaces, include pH and ionic strength.

The direct detection and visualization of adsorption of single NPs to the differently functionalized sensor surfaces enables ultrasensitive and unambiguous characterization of NP-surface interaction. Besides applications in analytics and basic research, this can be used for nanotoxicology studies. To achieve this aim, the adsorption of nanoparticles to biomimetic surfaces (e.g. coated by phosphatidylcholine and by polysaccharides from glycocalyx) can be determined and compared with toxicity of nanoparticles towards cell lines. Thus the nanotoxicity related Quantified Structure Activity Relationship (QSAR) for selected nanoparticles can be determined.

## Electronic supplementary material

Electronic supplementary material includes video sequences obtained by visualization of adsorption of 40 nm cit-Au NP (Video1.mp4) or 60 nm bPEI-Ag NP (Video2.mp4) to the patterned surface containing the spots coated by C11-N-(CH3)3 + Cl− -terminated alkylthiol while the rest of the surface was coated by C10-COOH -terminated alkylthiol. Each newly adsorbed NP is indicated by red circle, then its color is changed to the blue one. The red plot below shows the number of NPs adsorbed during the current frame, the blue plot shows the total number of adsorbed nanoparticles.

ESM 1 (MP4 1026 kb)

ESM 2 (MP4 2936 kb)
